# Athletes’ access to, attitudes towards and experiences of help-seeking for mental health: a scoping review

**DOI:** 10.1136/bmjopen-2024-097492

**Published:** 2025-08-07

**Authors:** Kirsty R Brown, Mary L Quinton, Grace Tidmarsh, Jennifer Cumming

**Affiliations:** 1 School of Sport, Exercise and Rehabilitation Sciences, University of Birmingham, Birmingham, UK

**Keywords:** MENTAL HEALTH, SPORTS MEDICINE, PSYCHIATRY, Systematic Review

## Abstract

**Abstract:**

**Objectives:**

Athletes have been found to experience a similar prevalence of mental health issues to non-athletes. However, they are subjected to a greater array of barriers to help-seeking for mental health, including sport-specific factors. This scoping review synthesised the literature on athletes’ access to, attitudes towards and experiences of help-seeking for mental health from formal (mental health professionals such as psychiatrists) and semiformal sources (those who are not mental health professionals but are a service provider such as a coach).

**Design:**

The Joanna Briggs Institute framework and recommendations were used alongside the Preferred Reporting Items for Systematic Reviews and Meta-Analyses-Protocols checklist for scoping reviews. This scoping review was predominantly informed by Arksey and O’Malley’s framework for scoping reviews, supplemented by Levac *et al*’s additional recommendations. Rickwood and colleagues’ help-seeking frameworks informed the research question, inclusion/exclusion criteria and analysis.

**Data sources:**

The search terms and synonyms of “athlete” AND “mental health” AND “help-seeking” were searched in PsychINFO, Embase, MEDLINE, APA PsychArticles Full Text, Web of Science Core Collection, Scopus, Sport Discus, CINAHL and Proquest (Education Database, Health & Medical Collection, Nursing & Allied Health database, Psychology Database, Public Health Database, Education Collection, and Medicine & Education). These searches were conducted at three time points between April 2022 and 2024.

**Eligibility criteria:**

The inclusion and exclusion criteria were initially predetermined and specified in the protocol paper published in BMJ Open. Primary research articles, interventions and systematic reviews that referred to semiformal and formal sources of support were included.

**Data extraction and synthesis:**

The lead reviewer (KRB) screened all titles and abstracts, and full texts, and extracted data from all included studies. A second reviewer was involved in screening and extracting 20%–30% of studies at each stage. Findings were synthesised descriptively (eg, study population, data collection method and location of studies) and by content (eg, access, attitudes and experiences, sources of support, use of theory and the validity of quantitative measures used).

**Results:**

After screening 4954 titles and abstracts and 275 full texts in Covidence, 104 papers were included in the review. This comprised of 87 primary research articles, 13 interventions and 4 systematic reviews. Most of the primary articles and interventions were published in the USA (50%). 49.4% of the primary articles used quantitative methods, 34.5% used qualitative methods and 16.1% used mixed methods. Attitudes towards mental health help-seeking were investigated in 78.8% of the included studies, experiences of help-seeking in 53.8% and access to sources of support in 31.7% of studies. Of the primary articles and interventions, formal sources were investigated in 55% of studies, semiformal sources in 2% and both in 26% of studies.

**Conclusions:**

This scoping review of 104 papers showed the benefit of using help-seeking frameworks to shape and analyse a review. Analysing the results using these frameworks provided a novel contribution to the literature, showing where the athlete help-seeking literature base is currently focused and identified gaps for further research. For example, there is a need for further research on athletes in less developed nations, more qualitative and mixed methods studies, and further research on athletes’ access to mental health support and their interactions with semiformal sources. The results have applied implications in public health and sport by highlighting the different factors that impact athlete help-seeking, and therefore areas where they require support.

**STRENGTHS AND LIMITATIONS OF THIS STUDY**

- A rigorous process was followed in the conduct of the scoping review by employing existing methodological frameworks, reviewing existing peer-reviewed scoping reviews and having four reviewers involved throughout the review process.
- This review has been informed by Rickwood and colleagues’ conceptual help-seeking frameworks, which provides a novel methodological contribution to the literature because athlete help-seeking research has previously lacked the use of theory.
- This scoping review only included articles referring to formal and semiformal sources of support and did not include articles that investigated athletes’ perspectives on informal sources of support and self-help, which are also likely to be used by athletes.
- This review only included peer-reviewed papers published in English and, therefore, could have missed relevant grey literature and papers in languages other than English.

## Introduction

### Background

 The prevalence of mental health conditions appears to be similar in athletes compared with the general population.^1^,^3^ What is less clear is the current state of the literature that encapsulates the perspective of athletes on different stages of the mental health help-seeking process. Mental health help-seeking is defined by Rickwood and Thomas (2012) as: ‘an adaptive coping process that is the attempt to obtain external assistance to deal with a mental health concern’^4^ (p180). A study by Edwards and Froehle (2023) found that irrespective of mental health diagnosis, treatment seeking was lower in athletes than non-athletes (23.0% in varsity athletes and 24.2% of club/intramural athletes compared with 31.1% of non-athletes had sought prior treatment).^3^ However, in a study comparing athletes to non-athletes in their eating disorder characteristics and rates of seeking treatment, there were no significant differences found in current treatment seeking and future intentions to seek help in those who screened positive for an eating disorder or threshold eating disorder.^5^ Evidently, a proportion of athletes are not getting the help required and face barriers towards seeking help for their mental health.

There are both sport-specific and general barriers to help-seeking for mental health in athletes. As discussed by Uphill *et al*,^6^ there are contextual factors associated with sport that can reduce the likelihood of an athlete seeking help. For example, athletes may fear that disclosure and help-seeking may have a negative impact on their selection to a team/squad.^7^ Furthermore, athletes may face difficulties distinguishing between mental health symptoms and physical symptoms that are expected to be experienced in sport, such as fatigue.^6 8^ Further barriers reported by Gulliver *et al* included stigma, a lack of knowledge of mental health services and negative past experiences.^8^ There is a need for a review on athlete help-seeking that encompasses these barriers and that is grounded in a help-seeking framework to ensure rigour and consistency across studies.

The current athlete help-seeking literature generally lacks the use of conceptual and theoretical frameworks,^4^ which makes comparisons across help-seeking studies difficult. Therefore, this review used Rickwood *et al*’s^9^ help-seeking framework and Rickwood and Thomas’^4^ conceptual measurement framework of help-seeking for mental health. These frameworks aided the formation of the research question, the aims of the review, exclusion and inclusion criteria and analysis of the data. The published protocol for this review maps the literature onto the different stages of these frameworks.^10^ Importantly for the purpose of this review, Rickwood and Thomas’ framework provided definitions for formal and semiformal sources of support. Formal support was defined as ‘professional health service providers with a specified role in the delivery of mental healthcare’. Examples include a ‘psychiatrist, psychologist, general practitioner, mental health nurse’ (p181). Comparatively, semiformal sources were defined as ‘service providers and professionals who do not have a specified role in the delivery of mental healthcare’. Examples include a ‘teacher, work supervisor, academic advisor, youth worker, coach’ (p181).

### Definitions of access, attitudes and experiences

The help-seeking frameworks^4 9^ also informed the definitions of access, attitudes and experiences alongside the theory of planned behaviour,^11^ knowledge of the results of qualitative studies and from discussions with the patient and public involvement (PPI) group. The reason for the formation of these definitions is provided in the methods. Access to seeking help refers to the availability of sources of help to the individual,^9^ including views on physical access to services (eg, the location of services). It is important to draw a distinction between access and attitudes when researching help-seeking as they encompass different factors.

Attitudes to help-seeking are focused on present views or future thoughts on seeking support (ie, future behavioural intentions to seek help^4^) including an individual’s willingness to disclose to sources in the present or future.^9^ Through the lens of the theory of planned behaviour, it is the ‘degree to which a person has a favourable or unfavourable’ view of seeking help for their mental health in the present moment, or in the future^11^ (p188). Attitudes to help-seeking also include perceptions of support meaning the view that individuals have on the role of these sources (ie, what does an individual think a source can help with). In another way, this can be understood as a person’s beliefs about support.^11^ Furthermore, attitudes encompass an individual’s preference for support when seeking help, including the personality characteristics of a source of mental health support and whether they are from within or outside the sport environment.^12^

Whereas attitudes refer to present or future views, experiences are focused on the help-seeking that an individual has previously engaged in. This is their past observable behaviour outlined within the process component of Rickwood and Thomas’ conceptual measurement framework.^4^ This conceptualisation includes help-seeking behaviour and whether an individual was willing to disclose to a source in the past.^9^ Finally, experiences comprise the comparison of past help-seeking behaviours between two groups (eg, comparing the help-seeking of athletes to non-athletes^13^) and studies that compare the number of athletes who actually seek help with the number who wanted to seek it (eg, 20% received support but 50% wanted it). The following section discusses these definitions in the context of current systematic reviews within athlete mental health, which informs the rationale for this study.

### Rationale

The current systematic reviews on the athlete help-seeking literature are limited and narrow in scope (ie, focused on barriers and facilitators to help-seeking).^14^,^17^ A systematic review by Moreland, Cox and Yang^16^ included 21 studies that synthesised the literature on barriers and facilitators to collegiate athletes’ use of mental health services. They also analysed the conceptualisation (ie, who the athlete was seeking help from and for what) and operationalisation (ie, details of the data collection method) of mental health service utilisation.^16^ Similarly, another systematic review by Castaldelli-Maia *et al*^15^ included 52 studies on barriers, facilitators, influencing factors and preferences for counsellor characteristics when help-seeking for mental health and also cultural influences on the mental health of elite athletes. Although both reviews synthesised the literature on barriers and facilitators to help-seeking for mental health, they only included studies on either elite athletes^15^ or collegiate athletes and their stakeholders (eg, coaches and athletic directors),^16^ which could exclude studies on athletes of mixed levels or recreational athletes. Although Moreland *et al*^16^ categorised the barriers and facilitators by personal characteristics, attitudes and opinions, and behaviour for both athletes and key stakeholders, these terms were not clearly defined and did not categorise barriers and facilitators by views on access. The latter is important to identify because without access to support, attitudes cannot be formed. In Castaldelli-Maia *et al*’s^15^ systematic review, beyond the separation of results into barriers, facilitators, influencing factors and counsellor characteristics, there was not a more detailed categorisation of these results (eg, into access, attitudes towards help-seeking or experiences). The categorisation of results into access, attitudes and experiences underpinned by a help-seeking framework is needed to advance the athlete help-seeking literature base.

Overall, the need for a more detailed analysis of athlete help-seeking studies and their results, including barriers and facilitators, informed the aims of the present scoping review. The categorisation of results is needed to aid the understanding of help-seeking in athletes and highlight which areas of the literature need further advancement. Therefore, this review investigated help-seeking in athletes and framed their results into athletes’ views on access, their attitudes towards help-seeking and their experiences of seeking help for their mental health. Given that there has been inconsistent use of theoretical frameworks to underpin previous reviews, it was also evident that the use of a conceptual help-seeking framework was important to shape the review and understand the results of studies on athlete help-seeking, which would also enable better comparisons between studies. A scoping review was an appropriate method for this topic as athlete mental health help-seeking within sport psychology is a relatively new area and evidence is continually emerging.^18^

### Objectives

The aims of this scoping review were to assess and map the literature on athletes’: (1) access to formal and semiformal sources of support, (2) attitudes towards and intentions to seek mental health support from formal and semiformal sources and (3) past experiences of interacting with formal and semiformal mental health support. In turn, the contributions of this review are to (1) highlight the current gaps in the athlete mental health help-seeking literature, (2) give suggestions for further research and (3) demonstrate the benefit of using help-seeking frameworks to shape and analyse the results of a scoping review.

## Methods

### Protocol, registration, and frameworks

This protocol was registered and published on medRxiv on the 21 February 2022^19^ and published in BMJ Open in 2023.^10^ Covidence^20^ was used to aid this review. The predominant methodological framework used for this scoping review was Arksey and O’Malley’s^21^ five-stage framework. The optional sixth stage of consultation was also employed in the form of PPI. To supplement this framework, other recommendations and frameworks were used. This included recommendations provided by Levac *et al*^18^ who supply further advice on each of the stages of Arksey and O’Malley’s framework. The Joanna Briggs Institute (JBI) framework and recommendations were used to ensure that the scoping review met its purpose and provided the Person-Concept-Context approach to inform the title of this scoping review.^22 23^ To ensure rigour, the Preferred Reporting Items for Systematic Reviews and Meta-Analyses Protocols (PRISMA-P) checklist (see online supplemental appendix 1) was used in the design and write-up stages of the review.^24^ Moreover, published scoping review protocols and result papers were consulted when designing this review.^25^,^32^ In addition to the methodological frameworks, this scoping review was informed by Rickwood *et al*’s.^9^ Help-seeking framework and Rickwood and Thomas’^4^ framework for mental health help-seeking. As will be shown, Rickwood and Thomas’^4^ framework informed the inclusion and exclusion criteria, and both informed the data extraction and analysis.

### Eligibility criteria

Stage 1 of Arksey and O’Malley’s framework is identifying the research question. The research question (‘Athletes access to, attitudes towards and experiences of help-seeking for mental health’) was informed by the JBI’s Population-Concept-Context framework alongside help-seeking frameworks provided by Rickwood and colleagues.^4 9 22^ The mapping of the research question onto these frameworks is provided in table 1 of the protocol paper.^10^

**Table 1:** Data charting process

Stage	Description of what was involved at this stage
1	Testing of the data extraction form by KRB and JC—five studies each.
2	Additional 20 papers independently extracted by KRB and JC to ensure that both had completed 20% of all included studies at this point.
3	The lead reviewer (KRB) went on to complete the consensus data extraction for 20% of papers.
4	Further changes to the data extraction form were made.
5	Formation of the definitions of access, attitudes and experiences.*
6	Decision to select access, attitudes and experiences, and formal and semiformal sources of support from the abstract, aims of the paper and data collection method.
7	The lead reviewer (KRB) completed all remaining data extractions independently.

* These definitions are provided in the introduction of this paper.

Stage 2 of Arksey and O’Malley’s framework is the identification of relevant studies including setting the eligibility criteria. As is recommended, the inclusion and exclusion criteria were predetermined and specified in the protocol paper.^10 18 21^ However, as the scoping review progressed, additional exclusion criteria were added at title and abstract and full-text screening to ensure that this review met its aims. This is to be expected in the ‘iterative’ process of conducting scoping reviews.^21^ These decisions were discussed between all reviewers (KRB, MLQ, JC and GT).^18^ Primary research articles, interventions and systematic reviews were included, whereas opinion pieces, magazine/newspaper articles and grey literature were excluded. Papers that referred to semiformal and formal sources of support were included, whereas papers on informal or self-help were excluded.^4^

Further exclusion criteria were added during screening. Studies were excluded if they only included data on the percentage of athletes that sought help. For example, a paper saying that ‘20% of athletes sought help for their mental health’ does not tell us anything about access, attitudes or experiences in relation to mental health help-seeking. However, if a paper compared athletes to non-athletes for rates of help-seeking or said that 20% wanted to seek help but only 10% did, then it was included as this indicates help-seeking experiences and attitudes. Additional exclusion criteria included at this stage were studies investigating physical injury (including concussions) and help-seeking, the relationship between social support and mental health or attitudes towards sport psychologists and/or not explicitly focused on help-seeking for mental health. Narrative reviews and conference proceedings and case studies were also excluded. The justifications for these additional eligibility criteria are provided in online supplemental appendix 2.

### Information sources and search

Initial searches and the identification of keywords and terms also fall within stage 2 of Arksey and O’Malley’s framework. Details of these stages are available in the protocol paper.^10^ The final search strategy including truncations and Boolean operators decided on was:

athlete AND mental health OR mental illness OR mental disorder OR well being OR wellbeing AND help seeking OR seeking help OR help OR treatment seeking OR seeking treatment OR support OR mental health service OR health service OR mental health care OR mental healthcare OR health care OR healthcare OR treatment seeking OR behavior OR help seeking behavior

The search strategy including limits applied and hits for each database is available in online supplemental appendix 3. The following databases were searched: PsychINFO (via OVID), Embase (via Ovid), MEDLINE (via Ovid), APA PsychArticles Full Text (via OVID), Web of Science Core Collection, Scopus, Sport Discus (via EBSCO), CINAHL (via EBSCO), Proquest (Education Database), Proquest (Health and Medical Collection), Proquest (Nursing and Allied Health database), Proquest (Psychology Database), Proquest (Public Health Database), Proquest (Education Collection), and Proquest (Sports Medicine & Education). These searches were first conducted between 30 March and 3 April 2022 and were rerun between 24 and 26 April 2023 and finally between 15 and 16 April 2024.

### Selection of sources of evidence

The lead reviewer (KRB) screened the first 70% of titles and abstracts before both reviewers (KRB and MQ) screened the remaining 30%. At this initial stage, the lead reviewer (KRB) selected ‘maybe’ for papers that needed further discussion, and these were screened again by both reviewers (KRB and MQ). Once all titles and abstracts had been screened, KRB and MQ met to resolve those that were marked as having a conflict, and a third reviewer was involved for those outstanding. For the updated search conducted in 2023, all additional titles and abstracts were screened by KRB, and MQ and either JC or GT were involved for required decisions. In total from the 2022, 2023 and 2024 searches, 4954 titles and abstracts were screened. By the end of screening for the searches conducted in 2022, 190 went for full-text review, which increased to 242 after the 2023 searches, and then to 275 after the 2024 searches.

Throughout the process of title and abstract screening, all reviewers kept in contact regarding decisions on inclusion and exclusion criteria to ensure transparency and meet recommendations that reviewers should meet at the beginning, middle and end of screening (Arksey & O’Malley, 2005; Levac *et al*, 2010; Peters *et al*, 2020). Of the 190 full texts from the 2022 searches, the lead reviewer (KRB) reviewed all full texts and one reviewer (MQ) reviewed 20%. Three reviewers (JC, MQ and KRB) met throughout the full-text screening to resolve any disagreements. For the additional full texts from the 2023 and 2024 search, the lead reviewer (KRB) screened and MQ, JC and GT were involved for any decisions needed. A lack of agreement meant that the paper was excluded. Following searches of reference lists during data extraction of the initially included studies, an additional nine papers were added to the review in 2022, and two in 2023 and one in 2024.

**Figure 1:**
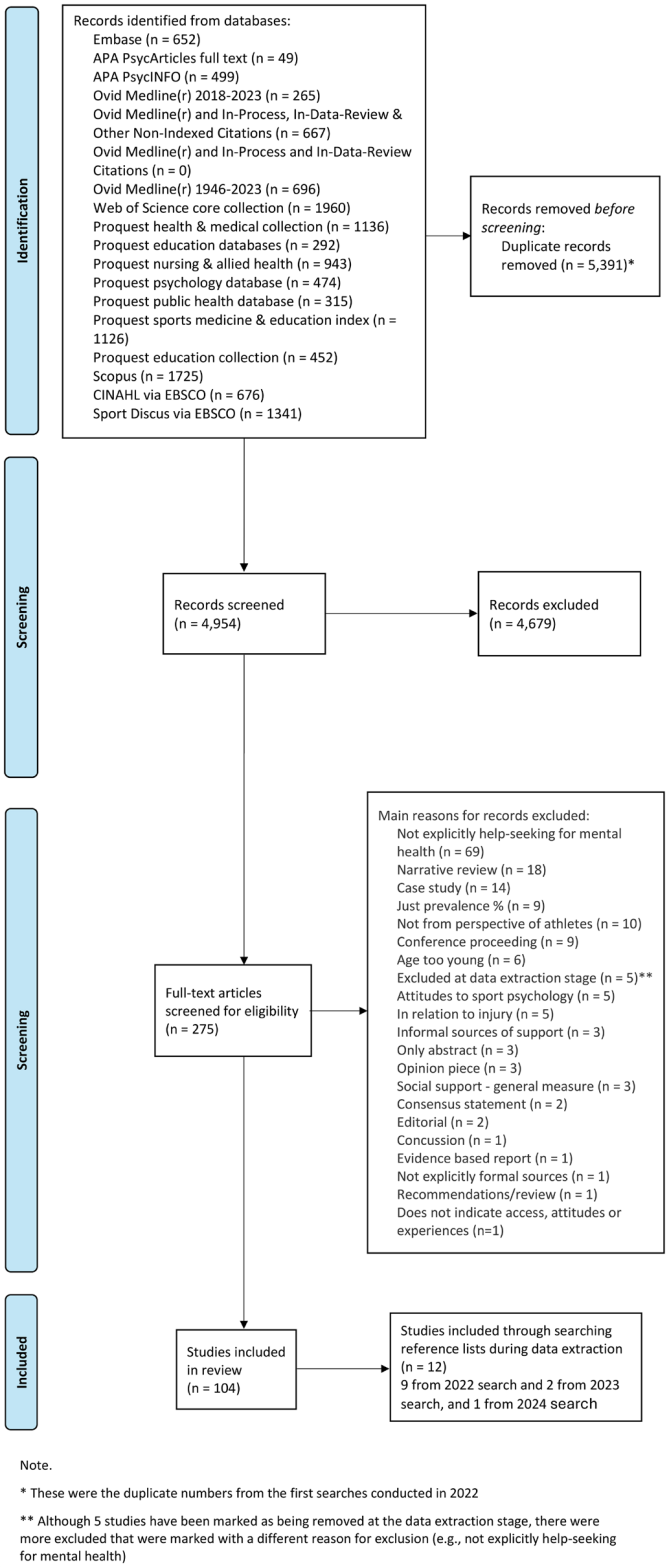
PRISMA flow diagram. PRISMA, Preferred Reporting Items for Systematic Reviews and Meta-Analyses.

During the data extraction stage, several studies were excluded for reasons such as the age of the participants being too young and the study not explicitly focusing on help-seeking for mental health. By the end of full-text screening, 104 papers were included in the review, as displayed in figure 1 (PRISMA flow diagram).

### Data charting process

Stage 4 of Arksey and O’Malley’s framework is charting the data. Covidence^20^ was used to create the data extraction form. The form for this review was influenced by other scoping reviews and the frameworks employed^1821^,^23 26 27 29 31^ as well as a data extraction form used for a systematic review that the lead reviewer (KRB) was previously involved in.^33^ Feedback was provided by JC, MQ and GT and amendments were made. Table 1 shows the steps involved in the data charting process. Further details of the data charting process and decisions are available in online supplemental appendix 4.

### Data items

The data items that were extracted are available in online supplemental appendix 5. Items included the year of publication, type of study and whether the study focused on access, attitudes or experiences.

### Synthesis of results

The data were analysed through: (1) descriptive numerical and (2) content analysis. The descriptive numerical analysis^18 21 22^ includes the overall number of studies, type of studies, year of publications, type of data collection method, study population and the number of studies from particular countries. In addition, directed content analysis was used to analyse the included studies, using existing theory and research to inform the analysis.^34 35^ Rickwood and colleagues’^4 9^ help-seeking frameworks informed the charting of the data, in the selection of access, attitudes and experiences, and formal and semiformal sources of support. All included studies were analysed with respect to their use of theory within the paper, and the use of validated help-seeking measures was analysed in quantitative and mixed methods studies. Details of studies and information extracted are available in online supplemental appendix 6.

### Patient and public involvement

An optional sixth stage of Arksey and O’Malley’s^21^ framework is consultation and stakeholder involvement and highlighted as important by Levac *et al*.^18^ PPI consisted of a group of six student-athletes and was used as consultation as part of a wider project that included this scoping review. They were first involved prior to the scoping review taking place to give us an understanding of the wider problem of mental health help-seeking in athletes. Student-athletes were appropriate as they are a subgroup of athletes at a risk of mental health difficulties owing to their dual career as a student and athlete.^36^ The PPI group informed the definitions of access, attitudes and experiences that were used in the analysis of papers included in the review. They were also consulted in the recommendations and further research directions that are provided as part of this paper, and ideas for dissemination that will follow from this publication such as feedback on blog posts that are written for the wider public and conference proceedings. The PPI group was not involved in the design, recruitment or conduct of the study.

## Results

### Characteristics and results of individual sources of evidence

The characteristics and results of sources of evidence are available in online supplemental appendix 6.

### Synthesis of results

Overview of included studies.

#### Type of articles included in the review and year of publication

**Figure 2:**
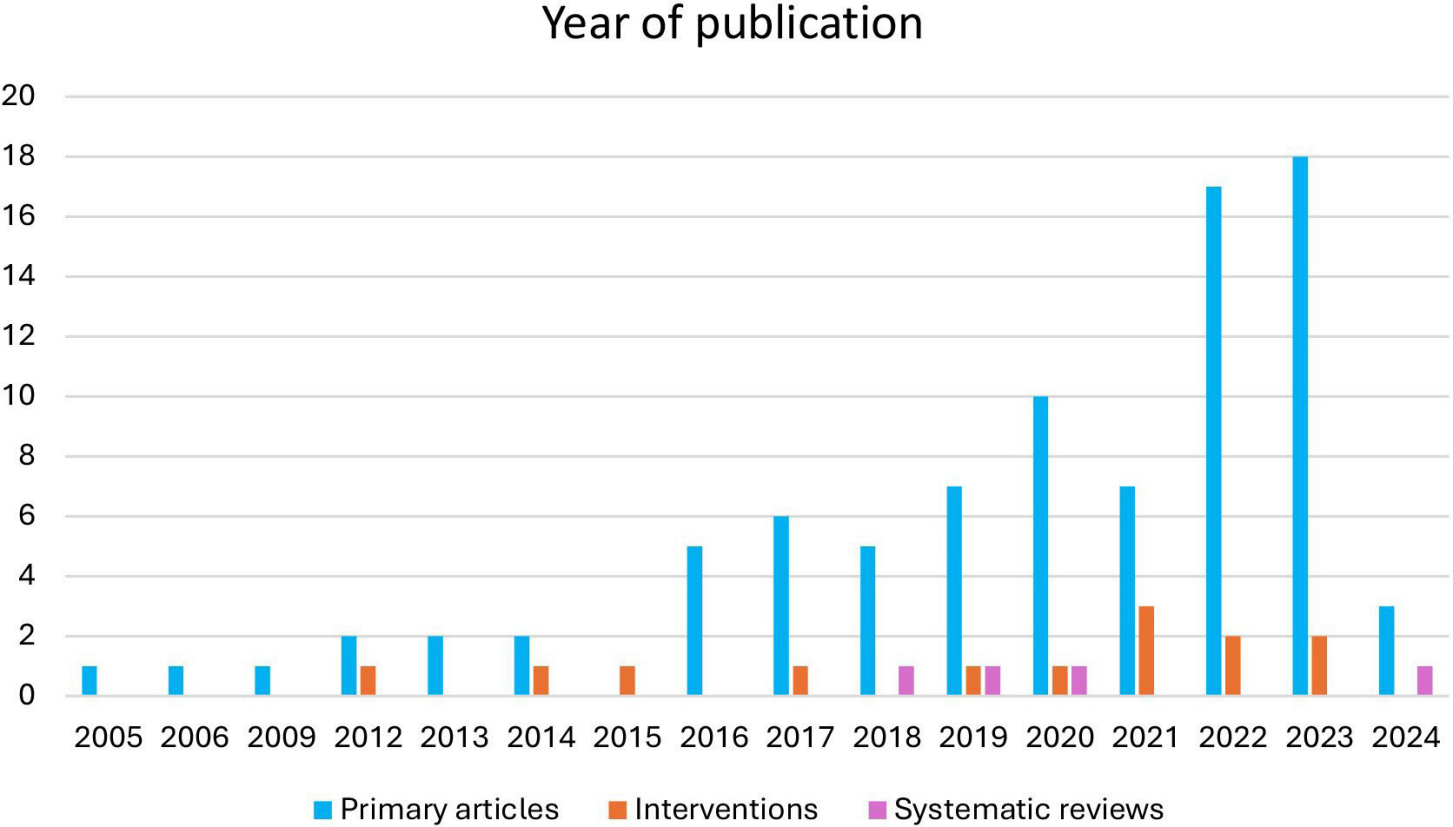
Year of publication of primary research articles, interventions and systematic reviews.

Of 104 included studies, there were 87 primary articles (83.7%), 13 interventions (13.0%) and 4 systematic reviews (3.8%). As shown in figure 2, the first article on athlete help-seeking was a primary research article published in 2005.^37^ From 2015, the number of primary articles increased until 2022. The primary articles in 2020, 2021, 2022, 2023 and 2024 accounted for 63.2% of the 87 primary articles. The first intervention study was published in 2012^38^ and the four systematic reviews were published in 2018, 2019, 2020 and 2024.^14^,^17^

#### Geographic location of included studies and the reporting of ethnicity

Most of the primary articles and interventions were conducted in the USA (50%), followed by the United Kingdom (UK)/Great Britain or England where 11% of the primary articles or interventions were conducted. Primary articles and interventions were conducted in 10 other countries including Ireland (n=7), Japan (n=4), Canada (n=5), Malaysia (n=1), Sweden (n=2) or a mix (n=8). Within the primary articles and interventions, 51 (51%) reported the ethnicity of athletes and 48% did not report any details of ethnicity. Further details are available in onlinesupplemental appendices 6^8^.

#### Population investigated in primary research articles and interventions and data collection method for primary articles

Student-athletes were the most investigated population in the athlete help-seeking literature, with 56% (n=56) of the 100 articles primary articles and interventions on this group. 33% (n=33) of the primary articles and interventions were on elite athletes. Elite athletes were either defined by the author or through interpretation of the study population by presented information; for example, ‘a member of Football Players Worldwide (FIFPRO) - the only global representative for professional football players’ (p2).^39^ In the remaining primary articles and intervention studies, eight included athletes of a mixed competitive level and three included athletes of a semi/subelite level (as defined by the author).

43 of the primary articles used quantitative methods of data collection (49.4% of the 87 primary articles). There were 30 qualitative articles (34.5% of primary articles). There were only 14 primary articles that used mixed methods (16.1%). Of these, only three articles used questionnaires and the use of a traditional qualitative method (eg, interviews) in combination^40^,^42^ and one used the Delphi technique.^43^

#### Use of validated measures in mixed methods or quantitative studies

Of both the quantitative and mixed methods primary articles and interventions, 51.4% of the studies included the use of a validated measure of help-seeking, whereas 48.6% did not. For those that did not, authors created their own measure or used an existing dataset such as the National College Health Assessment. Details are available in onlinesupplemental appendices 6  9.

### Content analysis

#### Access, attitudes and experiences towards help-seeking for mental health

Of all 104 included articles, attitudes were most frequently investigated (78.8%), followed by experiences (53.8%), and then access (31.7%). Further details of the individual studies that focused on access, attitudes or experiences are available in onlinesupplemental appendices 6  10. Of the primary articles, 77% investigated attitudes, 57.5% experiences and 32.2% access. Within intervention studies, 84.6% studied the impact of the intervention on attitudes towards help-seeking, 23.1% on experiences and 15.4% on access. Within these results, table 2 further outlines the number of studies that investigated the components in isolation or in various combinations, showing that access to help-seeking was the least investigated component in isolation (3.4%).

**Table 2:** Number of studies looking at access, attitudes and experiences of help-seeking

Component of help-seeking	Publications (n, %) (out of all 104)			Total
	Primary research articles	Interventions	Systematic reviews	
Access	3 (3.4)	1 (7.7)	0 (0)	4 (3.8)
Attitudes	25 (28.7)	8 (61.5)	0 (0)	33 (31.7)
Experiences	14 (16.1)	1 (7.7)	0 (0)	15 (14.4)
Access and attitudes	9 (10.3)	1 (7.7)	1 (25)	11 (10.6)
Access and experiences	2 (2.3)	0 (0)	0 (0)	2 (1.9)
Attitudes and experiences	19 (21.8)	2 (15.4)	1 (25)	22 (21.2)
Access, attitudes and experiences	13 (14.9)	0 (0)	2 (50)	15 (14.4)
Unclear	2 (2.3)	0 (0)	0 (0)	2 (1.9)
Total	87 (100)	13 (100)	4 (100)	104 (100)

To enable a more detailed analysis of studies that researched athletes’ attitudes towards help-seeking for mental health, subcategories were created based on the definition of attitudes towards help-seeking formed in the process of this review. This was extracted from quantitative and mixed methods studies that used validated help-seeking measures, and the selections made were based on these measures. Details are found in online supplemental appendix 6. Table 3 shows that general attitudes and stigma towards help-seeking were the most investigated in quantitative and mixed methods primary articles and interventions that used validated help-seeking measures, compared with preferences for support and behavioural intentions.

**Table 3:** Subcategorisation of attitudes in quantitative and mixed methods studies that use validated measures of help-seeking

	Subcategory of attitudes towards help-seeking			
	General attitudes	Stigma	Preferences for support	Behavioural intentions to seek help
Primary articles	17	15	10	10
Interventions	8	4	4	5
Total	25	19	14	15

These selections were informed by the validated measure(s) used in the study.

#### Formal and semiformal sources of support

Of all the primary articles and interventions, 81% researched help-seeking from formal sources of support and 28% investigated semiformal sources of support (available online supplemental appendix 10). Table 4 shows which of these studies looked at formal and semiformal sources in isolation or combined, and in which studies it was unclear from the abstract, aims or data collection method. Importantly, in over 50% of primary research articles and interventions, formal sources were investigated in isolation, whereas just two primary articles researched semiformal sources in isolation.

**Table 4:** Number of studies investigating formal and semiformal sources of support

Formal and/or semiformal source of support	Publications (n, %) (out of 100 primary articles and interventions)		
	Primary articles	Interventions	Total
Formal	46 (52.9)	9 (69.2)	55 (55)
Semiformal	2 (2.3)	0 (0)	2 (2)
Formal and semiformal	23 (26.4)	3 (23.1)	26 (26)
Unclear	16 (18.4)	1 (7.7)	17 (17)
Total	87 (100)	13 (100)	100 (100)

Formal and semiformal sources were only extracted from primary research articles and interventions.

To understand the impact of the use of validated versus non-validated help-seeking measures on the analysis of help-seeking access, attitudes, experiences and formal and semiformal sources of support, a pattern analysis was carried out. As displayed in table 5, quantitative and mixed methods studies that used validated help-seeking measures mostly investigated formal sources of support (69.4%) in isolation, but no studies that used validated measures investigated semiformal support in isolation. Validated measures were more explicit in what they measured in terms of formal and semiformal sources of support compared with non-validated measures. In 14.7% of primary articles and interventions that did not use validated measures, it was unclear if they were referring to formal or semiformal sources of support. Similarly, within the pattern analysis of validated measures by access, attitudes and experiences as shown in table 6, there were a lack of studies that used validated measures that measure access to help-seeking.

**Table 5:** Pattern analysis of validated and non-validated measures by formal and semiformal sources of support

	Validated measure(s) used			Non-validated measure(s) used		
	Primary articles	Interventions	Total	Primary articles	Interventions	Total
Formal	20 (71.4)	5 (62.5)	25 (69.4)	13 (44.8)	4 (80)	17 (50)
Semiformal	0 (0)	0 (0)	0 (0)	0 (0)	0 (0)	0
Formal and semiformal	8 (28.6)	3 (37.5)	11 (30.6)	12 (41.4)	0 (0)	12 (35.3)
Unclear	0 (0)	0 (0)	0 (0)	4 (13.8)	1 (20)	5 (14.7)
Total	28 (100)	8 (100)	36 (100)	29 (100)	5 (100)	34 (100)

Results are displayed as n (%).

**Table 6:** Pattern analysis of validated and non-validated measures by access, attitudes and experiences

	Validated measure(s) used			Not validated measure(s) used		
	Primary articles	Interventions	Total	Primary articles	Interventions	Total
Access	0 (0)	0 (0)	0 (0)	3 (10.3)	1 (20)	4 (11.8)
Attitudes	20 (71.4)	5 (62.5)	25 (69.4)	4 (13.8)	3 (60)	7 (20.6)
Experiences	1 (3.6)	0 (0)	1 (2.8)	10 (52.6)	1 (20)	11 (32.4)
Access and attitudes	4 (14.3)	1 (12.5)	5 (13.9)	0 (0)	0 (0)	0 (0)
Access and experiences	0 (0)	0 (0)	0 (0)	1 (3.4)	0 (0)	1 (2.9)
Attitudes and experiences	3 (10.7)	2 (25)	5 (13.9)	4 (13.8)	0 (0)	4 (11.8)
Access, attitudes and experiences	0 (0)	0 (0)	0 (0)	6 (20.7)	0 (0)	6 (17.6)
Unclear	0 (0)	0 (0)	0 (0)	1 (3.4)	0 (0)	1 (2.9)
Total	28 (100)	8 (100)	36 (100)	29 (100)	5 (100)	34 (100)

Results are displayed as n (%).

#### Use of theory to inform individual studies

Reflecting on the use of theory used to inform the studies within the review, only 21 of the 87 primary research articles used referred to a theoretical model or framework. Of these 21 studies, only eight used theory throughout the study to inform the background and discussion as well as the study design. Within the intervention studies, 6 of the 13 used theory, with five using theory throughout the study, including the intervention design. The details of theories used and in what detail in each individual study are found in online supplemental appendix 6.

## Discussion

### Summary of evidence

The aim of this scoping review was to assess and map the literature on athletes’ access to, attitudes and intentions, and experiences of interacting with semiformal and formal sources of support. In turn, this review aimed to highlight gaps in this literature, give suggestions for further research and show the benefit of using help-seeking frameworks to shape and analyse the results of a review.

#### Overview of included studies

Of the 104 articles included, there were significantly more primary research articles (n=87) than interventions (n=13). This disparity is understandable given that primary research provides evidence to support intervention development.^44 45^ It was apparent that all the primary articles and interventions were conducted in developed nations, with no relevant studies found in Africa or Latin America. Over half of the studies included were from the USA. In developed nations such as the USA, mental healthcare and access to services is often better than low and middle-income countries.^46^ Additionally, it was clear that this area of literature is dominated by a Western-centric perspective, prioritising these experiences and values. In contrast, there were limited studies from Asian nations, with four studies from Japan and one from Malaysia, despite the growing interest in athlete mental health in this region. Notably, two of the studies included in the review from Japan focused on rugby players.^47 48^ Despite Japan also being a developed country, healthcare and cultural aspects that impact mental health help-seeking will be unique and differ to Westernised nations.^49^ Importantly, across but also within regions, there will be differing levels of mental health stigma, access to services including different insurance systems and healthcare policies, which will all impact athletes’ help-seeking.^46 50 51^ We, therefore, call for more diverse research on mental health help-seeking in athletes, with a focus on the Global South very much needed. With respect to the subpopulation studied, 56% of the included primary articles and interventions were on student-athletes, with a significant number of these being US student-athletes. Similar results were found in a systematic scoping review of student-athlete mental health, whereby 67.3% of the 159 studies were on US student-athletes.^36^ In contrast, fewer studies focused on elite or semi/subelite athletes. For subpopulation analyses to be conducted in future, research will need to diversify the samples included.

This scoping review highlighted variation in the reporting of methodological details. Almost half of primary articles and interventions did not report any details on the ethnicity of athletes. In a recent literature review, a lack of reporting on ethnicity was also found in articles published in high-impact medical journals, with only 35% of the selected articles reporting ethnicity.^52^ Research indicates that ethnic minorities can experience greater stigma^53 54^ and reduced help-seeking,^55^,^57^ and therefore it is important to detail this demographic information. Within the quantitative studies, there was variance in the measures used with over 45% of the mixed methods and quantitative primary articles and interventions not using a validated help-seeking measure. It is evident that these findings are similar to a systematic review of the use of help-seeking measures in adolescent mental health research, whereby only 24% of studies used existing measures.^58^ The utilisation of different measures impacted the selection of access, attitudes and experiences and formal and semiformal sources of support in this review. In the pattern analysis of validated and non-validated measures by these constructs, it became evident that overwhelming more quantitative and mixed methods studies that used validated measures encapsulated athletes’ views on attitudes towards help-seeking and formal sources of support. The variety and lack of validated measures being used is an important finding to enhance the rigour of help-seeking research going forwards (see table 7 for future research recommendations).

**Table 7:** Future research directions and evidence from this review

Overview of included studies	
**Systematic review recommendations**	
The athlete help-seeking literature would benefit from further systematic reviews which encompass the more recent athlete help-seeking literature.	There is a lack of systematic reviews on the athlete help-seeking. 4 of the 104 included studies were systematic reviews published in 2018, 2019, 2020 and 2024.
We recommend a systematic review on the prevalence of help-seeking in athletes, including a quality appraisal of these studies.	In this review, studies that only encapsulated rates of mental health help-seeking in athletes were excluded.
We recommend a systematic review on the help-seeking measures used in the athlete mental health help-seeking literature, particularly on the validated measures used and how this impacts the consistency of findings across the literature on athlete help-seeking for mental health.	This review highlighted the variety of help-seeking measures used. Although a pattern analysis was carried out, a more detailed review of help-seeking measures in the athlete mental health literature is warranted.
**Study location**	
Research is required on athletes in less developed countries because research indicates that levels of mental health stigma may be greater in less developed countries than developed nations^50 71 72^ due to reasons such as cultural factors (eg, shame and power differences between therapists and patients.)^73^	Most of the studies were on athletes in developed and westernised nations.
There is a need for research on athletes in other westernised countries across Europe, such as the UK.	The USA made up half of the primary articles and interventions.
There is a need for research on athletes in across Asian countries (eg, Japan) and countries within Africa and Latin America that consider different contextual factors (eg, healthcare policies and stigma) that can impact athlete help-seeking.	There were only four included studies from Asian countries and none from African or Latin American countries.
**Ethnicity**	
Future research should include details on the ethnicity of athletes to allow comparisons to be made and to identify ethnic groups that may be more at risk of reduced help-seeking in athletes. This is important as research indicates that ethnic minorities can experience greater stigma^53 54^ and reduced help-seeking.^55^,^57^	There was a lack of reporting of ethnicity in studies and issues around the terminology used to describe ethnicity, often with the term ‘race’ used in replacement.
The research needs to specify race or ethnicity and how it is defined in their study to allow for comparisons between studies to be made.^74^	
**Methods**	
There is a need for more qualitative and mixed methods studies to investigate help-seeking in athletes to gain a more in-depth understanding of help-seeking experiences.	More of the studies included in this review were quantitative in nature than qualitative.
**Subpopulation**	
The perspective of individuals that interact with and provide mental health support to athletes is also important and this literature should be investigated in future reviews. Beyond the opinion of athletes’, literature has focused on the perspective of other individuals on supporting athletes’ mental health. For example, both Lebrun *et al*^75^ and Mazzer and Rickwood^76^ investigated the experiences of coaches and their perceived role in supporting young athletes mental health.	This review only investigated athletes’ perspectives on help-seeking.
**Content analysis of included papers**	
**Access, attitudes and experiences**	
Greater research is required on access to help-seeking in athletes because to understand attitudes towards and experiences of help-seeking, athletes must have access to help.	Access, attitudes and experiences for mental health help-seeking have all been covered within this literature base but access is the least investigated component.
Further research should also define what aspect of help-seeking they are investigating.	Definitions on the aspects of help-seeking investigated in included studies were lacking.
**Sources of support**	
It is evident that more research is required on athletes’ interactions with semiformal sources of support including the attitude that athletes have towards them, and experiences of interacting with them if they have done so. This is important to understand as semiformal sources of support (eg, coaches and academic tutors) may be more readily available to athletes and interact with them more frequently.	Only two studies looked at athletes’ perspectives on semiformal sources of support specifically.
Irrespective of the source of support investigated it is recommended that further research be clear in what source of support they are referring to. As mentioned in Rickwood and Thomas’^4^ help-seeking measurement framework, it is important to identify the source being investigated. Therefore, within the athlete help-seeking literature, the sources of support need to be better defined.	In 17% of primary articles and interventions the source of support being referred to was not clear from the abstract, aims or explanation of the data collection method.
**Theory**	
Further research should ensure they use existing theories and frameworks to understand athlete help-seeking. For example, motivational frameworks such as the theory of planned behaviour, self-determination theory and Rickwood and colleagues' help-seeking frameworks.^4 9^ This will ensure that comparisons between future research can be made more easily, and therefore the literature can be advanced more quickly.	The use of help-seeking theories and frameworks within the included studies was limited.
**Help-seeking measures**	
The field of sport psychology needs to reach a consensus on which validated athlete mental health help-seeking measures are to be used within this growing area of research.	A variety of measures were used in the quantitative studies, and not all were validated.
There is a need for the development or use of existing validated measures on help-seeking from semiformal sources of support, and those that encapsulate athletes’ views on access and experiences.	The pattern analysis of help-seeking measures showed a lack of validated measures on athletes’ views on access and semiformal sources of support.

#### Content analysis: the benefit of using help-seeking frameworks to shape and analyse the results

The content analysis of studies in the review was benefitted by using Rickwood and colleagues’ help-seeking frameworks.^4 9^ The use of these frameworks also highlights the importance of understanding the quantity of research that has investigated the different components of access, attitudes and experiences, and formal and semiformal sources of support to understand where future research needs to focus. Access was the least investigated component and very few studies explored access in isolation. In Way *et al*’s^59^ study, the student-athletes expressed a need for the availability of services specific to student-athletes. Attitudes was investigated the most frequently and on it’s own across the included studies and encompassed self-stigma and public stigma towards help-seeking.^60 61^ As indicated in Rickwood *et al*’s^9^ help-seeking framework, ‘availability of sources of help’ (step 3) predisposes ‘willingness to seek out and disclose to sources’ (step 4). Therefore, it is important that research is also focused on both access and attitudes, because even if an individual has a positive attitude towards help-seeking, they cannot seek said support if it is not accessible to them. Experiences were investigated in just over half of included studies, and this was commonly examined alongside attitudes. This component was explored in several qualitative and mixed methods primary articles, particularly through interviews. For example, in Åkesdotter *et al*’s^62^ study, they explored elite athletes’ help-seeking journeys through life story interviews. Similarly, Jewett *et al*^42^ explored the interactions between athletes and clinicians when they sought help. It is important to understand athletes’ experiences of help-seeking for mental health as this can elicit further information on access and attitudes, and how athletes’ opinions may have been formed, especially as negative past experiences serve as barrier to help-seeking.^8^

Overwhelmingly more studies investigated formal sources of support as evidenced by the frequent use of Fisher and Turner’s^63^ Attitudes Toward Seeking Professional Psychological Help Scale. Several studies referred to a specific formal source of support, such as experiences and attitudes towards counselling.^64^,^66^ Only two primary research articles investigated athletes’ help-seeking from semiformal sources, and these studies referred to coaches as a source of support.^67 68^ In sport athletes likely have access to many potential forms of semiformal support such as coaches, club mental health first aiders and sports managers, which could be leveraged to improve mental health help-seeking. It is evident from several studies that athletes have preferences towards both formal and semiformal sources of support. For example, in Bird *et al*’s study, 36.6% of the student-athletes expressed that they would seek help from a coach, 16.8% from an athletic trainer and 18.8% from a mental health professional as well as other informal sources of support.^65^ However, there is a need for further research on athletes’ views of specified semiformal sources of support prior to their utilisation in interventions.

Evidently, there was a lack of theory used by the individual studies included within the review. The lack of help-seeking theories or models for athletes has been recognised in other studies.^69 70^ Therefore, underpinning the analysis with Rickwood and colleagues’ frameworks has provided a novel contribution to the literature and^1^,^69 10 18^ shaped the avenues for further research, which will be discussed next.

### Limitations and future research directions

There are a few limitations to this scoping review. Only papers published in English were included owing to the reviewers involved. The quality of the articles was not assessed in keeping with the aims of a scoping review.^21^ Due to the number of studies included in the review and the aims of this scoping review, it was not feasible to also include results of studies beyond whether they referred to access, attitudes or experiences, and formal or semiformal sources of support. Table 7 highlights further research directions as grounded in the limitations and results of the review.

## Conclusion

This was the first scoping review conducted on the athlete mental health help-seeking literature, which was appropriate given the relatively new literature base and limited systematic reviews. This scoping review included 104 studies, which mapped the literature on athletes’ access to, attitudes towards and experiences of help-seeking for mental health from formal and semiformal sources of support. The benefit of utilising help-seeking frameworks to aid the conduct of a scoping review was highlighted. Overall, most literature was focused on attitudes towards help-seeking and formal sources of support. Half of the articles were conducted in the USA and student-athletes were the most investigated population group. Quantitative methods of data collection were the most popular, but not all studies used validated help-seeking measures. This scoping review has highlighted many future research directions including the need for more qualitative studies on athletes’ outside of the USA and greater research on athletes’ views on semiformal sources of support. Aligned with one of the functions of scoping reviews, topics for further systematic reviews were highlighted. Beyond research, there are several applied implications of the findings of this scoping review. There is a need for mental health help-seeking interventions specific to, and embedded within, sports organisations. In particular, these should focus on improving athletes’ access to mental health support. Sports organisations should leverage the existing resources such as providing mental health first aid training to sports staff and encouraging athletes’ use of existing mental health apps.

## Supplementary material

10.1136/bmjopen-2024-097492 online supplemental file 1

10.1136/bmjopen-2024-097492 online supplemental file 2

10.1136/bmjopen-2024-097492 online supplemental file 3

## Data Availability

All data relevant to the study are included in the article or uploaded as supplementary information.
