# Supplementary material for: Athletes’ access to, attitudes towards and experiences of help-seeking for mental health: a scoping review
Source: BMJ Open. 2025 Aug 7;15(8):e097492. doi: 10.1136/bmjopen-2024-097492 (PMC12336477; doi:10.1136/bmjopen-2024-097492)
Supplement: online supplemental file 1 [file bmjopen-15-8-s001.docx]

**Supplementary File 1**

PRISMA-P Checklist

This is attached as a separate document

**Supplementary File 2**
Additional Exclusion Criteria Included in the Review

| Additional exclusion criteria | Details & justification |
| --- | --- |
| Papers on physical injury | Papers that focused on physical injury and help-seeking were excluded unless it was specifically in relation to mental health help-seeking following injury (e.g., papers that looked at social support during rehabilitation from injury). This is a different research question and population group to what this reviewed intended to look at. |
| Percentage of athletes that sought help | Papers that just included data on the percentage of athletes that sought help were excluded. For example, a paper saying that “20% of athletes sought help for their mental health” does not tell us anything about access, attitudes or experiences in relation to mental health help-seeking. However, if a paper compared athletes to non-athletes for rates of help-seeking or said that 20% wanted to seek help but only 10% did then it was included as this indicates help-seeking experiences and attitudes. |
| Social support and mental health | Papers that looked at the relationship between social support and mental health were excluded these papers are not specifically focused on formal and semi-formal sources of support. |
| Not explicitly about help-seeking for mental health | We had to be strict and exclude papers that were not explicitly focused on help seeking for mental health. I found this difficult as wanted to include some of these papers as they were interesting. But if they were looking at mental health in athletes more generally and just included a small part on treatment then the paper was excluded as it was not explicitly focused on help-seeking. For example, only 1 or 2 athletes discuss support. |
| Papers on concussion | There were a significant number of papers on concussion that were returned at title and abstract screening. Unless the paper were to specify help-seeking for mental health following a concussion then it was excluded.  Despite some of the papers mentioning mental health symptoms they were excluded because it wasn’t specifically help seeking in relation to mental health.  This is focused on a separate research question and population group. |
| Narrative reviews, conference proceedings & case studies | Narrative reviews, conference proceedings and case studies were excluded as there either wasn’t enough information to extract or not systematic and rigorous enough as a method. |
| Attitudes towards sport psychologists | Papers that looked at attitudes towards sport psychologists were excluded because this is a separate body of literature which looks at athletes attitudes to interacting with sport psychologists for sport performance as well as mental health. They are not focused enough on mental health to be included in the review. However, this body of literature can certainly be used to inform the help-seeking literature (this point is discussed in the discussion section). |

**Supplementary File** 2
*Search Strategy Including Databases Searched, Search Queries Restrictions Applied and Number of Searches Returned*

|  | |  | **2022 search** | **2023 search** | **2024 search** |
| --- | --- | --- | --- | --- | --- |
| **Database** | **Search Query** | **Restrictions applied** | **Number of hits** | **Number of hits** | **Number of hits** |
| Embase (via OVID) | athlet*.ti,ab,kw,sh.  AND  mental health.ti,ab,kw,sh. OR mental illness*.ti,ab,kw,sh. OR mental disorder*.ti,ab,kw,sh. OR well being.ti,ab,kw,sh. OR wellbeing.ti,ab,kw,sh.  AND  help seeking.ti,ab,kw,sh. OR seeking help.ti,ab,kw,sh. OR help.ti,ab,kw,sh. OR treatment seeking.ti,ab,kw,sh. OR seeking treatment.ti,ab,kw,sh. OR support.ti,ab,kw,sh. OR mental health service*.ti,ab,kw,sh. OR health service*.ti,ab,kw,sh. OR mental health care.ti,ab,kw,sh. OR mental healthcare.ti,ab,kw,sh. OR health care.ti,ab,kw,sh. OR healthcare.ti,ab,kw,sh. OR treatment seeking behavior.ti,ab,kw,sh. OR help seeking behavior.ti,ab,kw,sh. | English language  Remove preprint records  Review, short survey, article, letter, note, editorial | 428 | 556 | 652 |
| APA PsychArticles Full Text (via OVID) | athlet*.ti,ab,kw.  AND  mental health.ti,ab,kw. OR mental illness*.ti,ab,kw. OR mental disorder*.ti,ab,kw. OR well being.ti,ab,kw. OR wellbeing.ti,ab,kw.  AND  help seeking.ti,ab,kw. OR seeking help.ti,ab,kw. OR help.ti,ab,kw. OR treatment seeking.ti,ab,kw. OR seeking treatment.ti,ab,kw. OR support.ti,ab,kw. OR mental health service*.ti,ab,kw. OR health service*.ti,ab,kw. OR mental health care.ti,ab,kw. OR mental healthcare.ti,ab,kw. OR health care.ti,ab,kw. OR healthcare.ti,ab,kw. OR treatment seeking behavior.ti,ab,kw. OR help seeking behavior.ti,ab,kw. |  | 24 | 34 | 49 |

**Supplementary File** 3 **continued**

| APA PsychINFO (via OVID) | athlet*.ti,ab,id,sh.  AND  mental health.ti,ab,id,sh. OR mental illness*.ti,ab,id,sh. OR mental disorder*.ti,ab,id,sh. OR well being.ti,ab,id,sh. OR wellbeing.ti,ab,id,sh.  AND  help seeking.ti,ab,id,sh. OR seeking help.ti,ab,id,sh. OR help.ti,ab,id,sh. OR treatment seeking.ti,ab,id,sh. OR seeking treatment.ti,ab,id,sh. OR support.ti,ab,id,sh. OR mental health service*.ti,ab,id,sh. OR health service*.ti,ab,id,sh. OR mental health care.ti,ab,id,sh. OR mental healthcare.ti,ab,id,sh. OR health care.ti,ab,id,sh. OR healthcare.ti,ab,id,sh. OR treatment seeking behavior.ti,ab,id,sh. OR help seeking behavior.ti,ab,id,sh. | Peer reviewed  English language  Journal, peer-reviewed journal or peer-reviewed status unknown | 347 | 416 | 499 |
| --- | --- | --- | --- | --- | --- |
| Medline:   1. Ovid MEDLINE(R) 2017-2022* 2. Ovid MEDLINE(R) In-Process, In-Data-Review & Other Non-Indexed Citations 1946-2022* 3. Ovid MEDLINE(R) In-Process and In-Data-Review 1946-2022* 4. Ovid MEDLINE(R) 1946-2022*   (via OVID) | athlet*.ti,ab,kw,sh.  AND  mental health.ti,ab,kw,sh. OR mental illness*.ti,ab,kw,sh. OR mental disorder*.ti,ab,kw,sh. OR well being.ti,ab,kw,sh. OR wellbeing.ti,ab,kw,sh.  AND  help seeking.ti,ab,kw,sh. OR seeking help.ti,ab,kw,sh. OR help.ti,ab,kw,sh. OR treatment seeking.ti,ab,kw,sh. OR seeking treatment.ti,ab,kw,sh. OR support.ti,ab,kw,sh. OR mental health service*.ti,ab,kw,sh. OR health service*.ti,ab,kw,sh. OR mental health care.ti,ab,kw,sh. OR mental healthcare.ti,ab,kw,sh. OR health care.ti,ab,kw,sh. OR healthcare.ti,ab,kw,sh. OR treatment seeking behavior.ti,ab,kw,sh. OR help seeking behavior.ti,ab,kw,sh. | English language | 1. 152 2. 421 3. 4 4. 317 | 1. 199 2. 534 3. 1 4. 385 | 1) 265  2) 667  3) 0  4) 696 |

**Supplementary File** 4 **continued**

| Web of Science Core Collection | athlet*  AND  mental health OR mental illness* OR mental disorder* OR well being OR wellbeing  AND  help seeking OR seeking help OR help OR treatment seeking OR seeking treatment OR support OR mental health service* OR health service* OR mental health care OR mental healthcare OR health care OR healthcare OR treatment seeking behavior OR help seeking behavior | Topic search (TS=)  Exclude: book chapters, meeting abstracts & corrections | 1,245 | 1,567 | 1,960 |
| --- | --- | --- | --- | --- | --- |
| Proquest:   1. Health & Medical Collection 2. Education Database 3. Nursing & Allied Health 4. Psychology Database 5. Public Health Database 6. Sports Medicine & Education Index 7. Education Collection | athlet*  AND  mental health OR mental illness* OR mental disorder* OR well being OR wellbeing  AND  help seeking OR seeking help OR help OR treatment seeking OR seeking treatment OR support OR mental health service* OR health service* OR mental health care OR mental healthcare OR health care OR healthcare OR treatment seeking behavior OR help seeking behavior | Peer reviewed  Scholarly journals  English language | 1. 736 2. 198 3. 610 4. 338 5. 215 6. 833 7. 316 | 1. 923 2. 234 3. 758 4. 402 5. 273 6. 964 7. 364 | 1. 1,136 2. 292 3. 943 4. 474 5. 315 6. 1,126 7. 452 |
| CINAHL (via EBSCO) | athlet* OR athlete  AND  mental health OR mental illness* OR mental disorder* OR well being OR wellbeing  AND  help seeking OR seeking help OR help OR treatment seeking OR seeking treatment OR support OR mental health service* OR health service* OR mental health care OR mental healthcare OR health care OR healthcare OR treatment seeking behavior OR help seeking behavior | Peer reviewed  Academic journals  English language | 484 | 566 | 676 |

**Supplementary File** 5 **continued**

| Sport Discus (via EBSCO) | athlet* OR athlete  AND  mental health OR mental illness* OR mental disorder* OR well being OR wellbeing  AND  help seeking OR seeking help OR help OR treatment seeking OR seeking treatment OR support OR mental health service* OR health service* OR mental health care OR mental healthcare OR health care OR healthcare OR treatment seeking behavior OR help seeking behavior | Peer reviewed  Academic journals  English language | 721 | 865 | 1,341 |
| --- | --- | --- | --- | --- | --- |
| Scopus | athlet*  AND  “mental health” OR mental illness* OR mental disorder* OR “well being” OR “wellbeing”  AND  “help seeking” OR “seeking help” OR “help” OR “treatment seeking” OR “seeking treatment” OR “support” OR mental health service* OR health service* OR “mental health care” OR “mental healthcare” OR “health care” OR “healthcare” OR “treatment seeking behavior” OR “help seeking behavior” | TITLE-ABS-KEY  Document type: article, review, editorial, short survey, note, letter  Publication stage: final  Language: English  Source type: Journal | 1,103 | 1,426 | 1,725 |

*Note*. Each search term was searched individually and then combined within the database. E.g. 1 OR 2 OR 3, or 17 AND 18 AND 19

**Supplementary File 4**

Data extraction process

| 1 | Testing of the data extraction form by KRB and JC – 5 studies each | Three reviewers (KRB, JC and MQ) met to discuss the data charting form – clarifications and amendments made. |
| --- | --- | --- |
| 2 | Additional 20 papers independently extracted by KRB and JC to ensure that both had completed 20% of all included studies at this point. | KRB compared extractions in Covidence and both reviewers (KRB and JC) met for clarifications. The third reviewer (MQ) was involved where required. Of importance, it was decided that mixed methods was to be selected if a survey included the use of an open written question alongside quantitative questions, as the open question was deemed to be the qualitative component. |
| 3 | The lead reviewer (KRB) went on complete the consensus data extraction for 20% of papers. | It became evident that the data extraction form required further changes. |
| 4 | Changes to the data extraction form were made. | All authors (KRB, JC, MQ, and GT) met to discuss review aims and results that would likely be presented to align with these aims. |
| 5 | Formation of the definitions of access, attitudes and experiences | It became apparent that it was difficult to ascertain the differences between access, attitudes and experiences and the results in relation to them. The definitions of access, attitudes and experiences were formed.* |
| 6 | Decision to select access, attitudes, and experiences, and formal and semi-formal sources of support from the abstract, aims of the paper and data collection method. | It was marked unclear if it was not explicit from these parts (the paper was still included as at full text screening it was deemed relevant and included in the results).  Both attitudes and experiences were selected if it was clear that the athletes’ current attitudes were shaped by their past experiences.  If a semi-formal or formal source of support was included in a measure that also referred to informal sources or self-help, then formal or semi-formal was selected based upon the sources included in the measures, and the informal source or self-help was ignored. To check if a data collection method (i.e., survey or questionnaire) included reference to formal and semi-formal sources, the details of this measure were sought for clarification.  In interventions access, attitudes, and experiences were selected based upon the goal of the intervention (i.e., is access, attitudes, or experiences towards help-seeking for mental health trying to be improved as a result of the intervention?). Formal or semi-formal was selected if the intervention was focused on improving mental health help-seeking from formal or semi-formal sources. |
| 7 | The lead reviewer (KRB) completed all remaining data extractions independently. | All other authors (JC, MQ and GT) were involved if clarifications were required.  Authors of papers were also emailed if further information or clarification was required. |

Note. *These definitions, and how they were formed are provided in the introduction.

**Supplementary File 5**

Data Extraction Form

| *Title of paper / abstract / report that data are extracted from* |
| --- |
| *Authors (Include corresponding author & email address)* |
| *Year of publication* |
| *Type of study (Systematic review, scoping review, primary research article, intervention)* |
| *Aims of the study* |
| *Type of study (Primary research article, intervention, systematic/scoping review)* |
|  |
| **Primary research article:** |
| *Location of study participants (e.g. country, city, institution)* |
| *Size of study population* |
| *Details of study population (description & details)*   - Age (mean & SD) - Gender (number & %) - Sport - mix or single sport (name single sport if applicable) - Level of competition (e.g. elite, university) (if applicable number & %) - Ethnicity (number & %) |
| *Data collection method (qualitative, quantitative, mixed methods)* |
| *Do they focus on access, attitudes or experiences? (tick box)*   - Access (incl. knowledge of access, physical access) - Attitudes (incl. perspectives) - Experiences |
| *Is the paper referring to formal or semi-formal sources of support?*  Formal sources: e.g. university counsellors & welfare officers (higher education), & GP, psychologist and psychiatrist (healthcare)  Semi-formal: e.g. university lecturer, academic tutor (higher education), sports coach, sports manager (in sport context), physiotherapist & dietitian (healthcare) |
| *Details of data collection method (in relation to access, attitudes or experiences)*   - For quantitative: questionnaires used & outcomes of interest - Just state if qualitative |
|  |
| **Systematic/scoping review** |
| *Number of studies included* |
| *Results*  Does the review focus on access, attitudes or experiences? (Just state, no details) |
| *Quality assessment*  Did the authors do a quality assessment?   - Yes - No |
|  |
| **Intervention Studies** |
| *Location of study (e.g. country, city, institution)* |
| *Study population:*   - Number & percentage of patients allocated to intervention - Number and percentage of participants allocated to comparator/control group |
| *Study population (for intervention group only)*   - Age (mean & SD) - Gender (number & %) - Sport (mix or specific sport if applicable) -  *not as much detail as originally required* - Level of competition (e.g. elite, university) (if applicable number & %) - Ethnicity (number & %) |
| *Description of intervention/exposure*   - What source of support are they referring to in their intervention? E.g. semi-formal or formal sources of support? |
| *Was there a comparison group? (Yes or no)* |
| *Goals & outcomes*  Is the intervention trying to improve help-seeking in relation to access, attitudes or experiences? (No details just state)    What are the main outcome(s) of the intervention & how is it measured? (Just state) |
|  |
| *Reference List*  Other studies of interest for the review indicated in the study reference list |

**Supplementary File 6**
Spreadsheet of all Papers and Raw Extracted Data

This is attached as a separate excel file.

*Note.* There may be differences in the data extracted owing to changes in the data extraction form as discussed in the methods section of the scoping review.

**Supplementary File 7**
Geographic Location of Included Studies

| Geographic location (country) | Publications (n, %) | | |
| --- | --- | --- | --- |
|  | Primary research articles | Interventions | Total |
| Australia | 3 (3.4) | 2 (15.4) | 5 (5) |
| Canada | 5 (5.7) | 0 (0) | 5 (5) |
| England | 2 (2.3) | 0 (0) | 2 (2) |
| Germany | 2 (2.3) | 0 (0) | 2 (2) |
| Iceland | 1 (1.1) | 0 (0) | 1 (1) |
| Ireland | 6 (6.9) | 1 (7.7) | 7 (7) |
| Japan | 3 (3.4) | 1 (7.7) | 4 (4) |
| Malaysia | 1 (1.1) | 0 (0) | 1 (1) |
| Malta | 1 (1.1) | 0 (0) | 1 (1) |
| Aotearoa New Zealand | 1 (1.1) | 0 (0) | 1 (1) |
| Sweden | 2 (2.3) | 0 (0) | 2 (2) |
| UK or Great Britain | 8 (9.2) | 2 (15.4) | 9 (9) |
| USA | 45 (51.7) | 5 (38.5) | 50 (50) |
| Mix | 7 (8.0) | 1 (7.7) | 8 (8) |
| Unclear | 0 (0) | 1 (7.7) | 1 (1) |
| Total | 87 (100) | 13 (100) | 100 (100) |

**Supplementary File 8**
The Reporting of Ethnicity in Primary Articles and Interventions Included in the Review

| Ethnicity reported | Publications (n, %) | | |
| --- | --- | --- | --- |
|  | Primary articles | Interventions | Primary articles + interventions |
| Yes (with statistics) | 43 (49.4) | 5 (38.5) | 48 (48) |
| Yes (no statistics) | 3 (3.4) | 0 (0) | 3 (3) |
| No | 40 (46.0) | 8 (61.5) | 48 (48) |
| Unclear | 1 (1.1) | 0 (0) | 1 (1) |
| Total | 87 (100) | 13 (100) | 100 (100) |

**Supplementary File 9**

The Use of Validated Measures in Mixed Methods and Quantitative Primary Research Articles and Interventions

| Use of a Validated Measure or not | Publications (n, %) (Out of 70 Mixed Methods or Quantitative Primary Articles and Interventions) | | |
| --- | --- | --- | --- |
|  | Primary Articles | Interventions | Total |
| Includes the use of validated quantitative measure of help-seeking | 28 (49.1) | 8 (61.5) | 36 (51.4) |
| Made their own measure of help-seeking or use of large survey data | 29 (50.9) | 5 (38.5) | 34 (48.6) |
| Total | 57 (100) | 13 (100) | 70 (100) |

*Note.* Details of the data collection methods for each study can be found in Supplementary File 6.

**Supplementary File 10**

Content Analysis of Included Studies

| Author (s), Year and totals | Access | Attitudes | Experiences | Formal | Semi-formal |
| --- | --- | --- | --- | --- | --- |
|  | Primary research articles | | | | |
| (Abela et al., 2021) |  | ✓ | ✓ | ✓ |  |
| (Agnew et al., 2018) |  |  | ✓ | - | - |
| (Åkesdotter et al., 2023) |  | ✓ | ✓ | ✓ |  |
| (Åkesdotter et al., 2020) | - | - | - | ✓ |  |
| (Anchuri et al., 2020) |  |  | ✓ | ✓ | ✓ |
| (Barnard, 2016) |  | ✓ |  | ✓ |  |
| (Biggin et al., 2017) |  | ✓ |  | ✓ | ✓ |
| (Bird et al., 2020) | ✓ | ✓ | ✓ | ✓ |  |
| (Bird et al., 2018) | ✓ | ✓ |  | ✓ | ✓ |
| (Bird et al., 2021) |  | ✓ |  | ✓ |  |
| (Bissett & Tamminen, 2022) |  | ✓ | ✓ |  | ✓ |
| (Crawford et al., 2023) | ✓ | ✓ | ✓ | - | - |
| (Cutler & Dwyer, 2020) |  | ✓ |  | ✓ | ✓ |
| (R. M. Daltry et al., 2023) |  |  | ✓ | ✓ | ✓ |
| (R. Daltry et al., 2018) |  | ✓ | ✓ | ✓ |  |
| (Delenardo & Terrion, 2014) |  | ✓ | ✓ | - | - |
| (Dixon et al., 2022) |  | ✓ |  |  | ✓ |
| (Drew & Matthews, 2019) |  |  | ✓ | ✓ | ✓ |
| (Edwards & Froehle, 2023a) |  |  | ✓ | ✓ | ✓ |
| (Edwards & Froehle, 2023b) |  |  | ✓ | ✓ |  |
| (Edwards et al., 2023) |  | ✓ | ✓ | - | - |
| (Edwards et al., 2022) |  | ✓ | ✓ | ✓ |  |
| (Fawver et al., 2024) |  |  | ✓ | ✓ |  |
| (Foong & Kwan, 2021) | - | - | - | - | - |

**Supplementary File 10 Continued**

| Author (s), Year and totals | Access | Attitudes | Experiences | Formal | Semi-formal |
| --- | --- | --- | --- | --- | --- |
|  | Primary research articles | | | | |
| (Giovannetti et al., 2019) | ✓ | ✓ | ✓ | ✓ | ✓ |
| (Gulliver, Griffiths, & Christensen, 2012) | ✓ | ✓ | ✓ | ✓ |  |
| (Habeeb et al., 2022) |  | ✓ |  | ✓ | ✓ |
| (Harris & Maher, 2022) | ✓ | ✓ | ✓ | ✓ |  |
| (Harrison & Buscombe, 2023) | ✓ | ✓ | ✓ | ✓ | ✓ |
| (Hatteberg, 2020) |  | ✓ | ✓ | - | - |
| (Hilliard et al., 2019a) |  | ✓ |  | ✓ |  |
| (Hilliard et al., 2019b) |  | ✓ |  | ✓ |  |
| (Hilliard et al., 2022) |  | ✓ |  | ✓ |  |
| (Hopley et al., 2022) | ✓ |  | ✓ | ✓ |  |
| (Jewett et al., 2021) |  | ✓ | ✓ | ✓ |  |
| (T. V. Jones, 2016) |  | ✓ |  | ✓ |  |
| (Junge & Prinz, 2019) |  |  | ✓ | ✓ |  |
| (Kilcullen et al., 2022) |  |  | ✓ | ✓ |  |
| (King et al., 2023) | ✓ | ✓ | ✓ | ✓ |  |
| (Kola-Palmer et al., 2020) | ✓ | ✓ | ✓ | ✓ | ✓ |
| (Kraus & Tibbetts, 2022) | ✓ | ✓ | ✓ | ✓ | ✓ |
| (Kroshus, 2017) |  | ✓ |  | ✓ |  |
| (Lane et al., 2023) |  |  | ✓ | - | - |
| (Leimer et al., 2014) |  | ✓ | ✓ | ✓ |  |
| (Lõpez & Levy, 2013) |  | ✓ |  | ✓ |  |
| (Lyons et al., 2024) |  | ✓ | ✓ | ✓ |  |
| (Marsters et al., 2021) |  | ✓ |  | ✓ | ✓ |
| (Martin & Anderson, 2020) |  | ✓ |  | ✓ | ✓ |
| (McArdle & Moore, 2013) | ✓ | ✓ | ✓ | ✓ | ✓ |

**Supplementary File 10 Continued**

| Author (s), Year and totals | Access | Attitudes | Experiences | Formal | Semi-formal |
| --- | --- | --- | --- | --- | --- |
|  | Primary research articles | | | | |
| (McGraw et al., 2018) |  |  | ✓ | - | - |
| (McLoughlin et al., 2023) |  | ✓ | ✓ | ✓ |  |
| (Miller et al., 2024) | ✓ | ✓ | ✓ | - | - |
| (M. Moore, 2017) |  | ✓ |  | ✓ |  |
| (M. A. Moore, 2016) | ✓ |  |  | ✓ |  |
| (Neumann et al., 2023) |  | ✓ |  | ✓ | ✓ |
| (Noguchi et al., 2022) | ✓ | ✓ | ✓ | - | - |
| (O’Keeffe et al., 2022) | ✓ | ✓ | ✓ | ✓ |  |
| (O’Keeffe, O’Connor, et al., 2023) | ✓ | ✓ |  | ✓ |  |
| (Oftadeh-Moghadam & Gorczynski, 2022) | ✓ | ✓ |  | ✓ | ✓ |
| (Ogden et al., 2023) |  | ✓ | ✓ | - | - |
| (Oguro et al., 2023) |  | ✓ | ✓ | ✓ | ✓ |
| (Ojio et al., 2021) |  | ✓ |  | ✓ |  |
| (Perry et al., 2022) | ✓ | ✓ |  | ✓ | ✓ |
| (Plateau et al., 2017) |  | ✓ | ✓ | ✓ |  |
| (Poucher et al., 2023) | ✓ |  | ✓ | - | - |
| (Prinz et al., 2016) |  |  | ✓ | ✓ |  |
| (Ramaeker & Petrie, 2019) |  | ✓ |  | ✓ |  |
| (Ryan et al., 2022) | ✓ | ✓ |  | - | - |
| (Sandgren et al., 2023) |  | ✓ | ✓ | - | - |
| (Sasso et al., 2022) |  | ✓ | ✓ | ✓ | ✓ |
| (Steinfeldt & Steinfeldt, 2012) |  | ✓ |  | ✓ |  |
| (Steinfeldt et al., 2009) |  | ✓ |  | ✓ |  |
| (Tabet et al., 2021) |  | ✓ |  | ✓ |  |
| (Tahtinen & Kristjansdottir, 2019) |  | ✓ | ✓ | ✓ |  |
| **Supplementary File 10 Continued** | | | | | |
| Author (s), Year and totals | Access | Attitudes | Experiences | Formal | Semi-formal |
|  | Primary research articles | | | | |
| (Tran, 2022) | ✓ | ✓ | ✓ | ✓ |  |
| (Van Ramele et al., 2017) | ✓ |  |  | - | - |
| (Wahto et al., 2016) |  | ✓ |  | ✓ |  |
| (Wasylkiw & Clairo, 2018) |  | ✓ |  | ✓ |  |
| (D. R. Watson et al., 2023) |  | ✓ |  | ✓ |  |
| (J. C. Watson, 2005) |  | ✓ |  | ✓ |  |
| (J. C. Watson, 2006) | ✓ | ✓ |  | ✓ |  |
| (Way et al., 2020) | ✓ |  |  | ✓ | ✓ |
| (Wilkerson et al., 2020) |  | ✓ | ✓ | ✓ |  |
| (Wilkins et al., 2020) | ✓ | ✓ |  | ✓ |  |
| (Wood et al., 2017) |  |  | ✓ | - | - |
| (Yoon & Petrie, 2023) | ✓ | ✓ | ✓ | ✓ | ✓ |
| (Young et al., 2022) | ✓ | ✓ | ✓ | ✓ | ✓ |
| Total for primary articles (n, %): | 28 (32.2) | 67 (77) | 50 (57.5) | 69 (79.3) | 25 (28.7) |
|  | Intervention studies | | | | |
| (Beauchemin, 2014) |  | ✓ |  | ✓ |  |
| (Chow et al., 2021) | ✓ | ✓ |  | ✓ |  |
| (Confectioner et al., 2021) |  | ✓ |  | ✓ | ✓ |
| (Donachie & Hill, 2022) |  | ✓ |  | ✓ |  |
| (Donohue et al., 2021) |  | ✓ |  | ✓ |  |
| (Gulliver, Griffiths, Christensen, et al., 2012) |  | ✓ | ✓ | ✓ |  |
| (C. Jones et al., 2022) |  | ✓ |  | ✓ | ✓ |
| (Kaneko et al., 2019) |  |  | ✓ | ✓ |  |
| (Kern et al., 2017) |  | ✓ |  | - | - |

**Supplementary File 10 Continued**

| Author (s), Year and totals | | Access | Attitudes | Experiences | Formal | Semi-formal |
| --- | --- | --- | --- | --- | --- | --- |
|  | Intervention studies | | | | |  |
| (Martin et al., 2020) | |  | ✓ | ✓ | ✓ | ✓ |
| (O’Keeffe, Chéilleachair, et al., 2023) | |  | ✓ |  | ✓ |  |
| (Oftadeh-Moghadam et al., 2023) | |  | ✓ |  | ✓ |  |
| (Van Raalte et al., 2015) | | ✓ |  |  | ✓ |  |
| Total for interventions (n, %): | | 2 (15.4) | 11 (84.6) | 3 (23.1) | 12 (92.3) | 3 (23.1) |
|  | Systematic reviews | | | | |  |
| (Bu et al., 2020) |  | ✓ | ✓ | X* | X* |  |
| (Castaldelli-Maia et al., 2019) | ✓ | ✓ | ✓ | X* | X* |  |
| (Cosh et al., 2024) | ✓ | ✓ | ✓ | X* | X* |  |
| (Moreland et al., 2018) | ✓ | ✓ |  | X* | X* |  |
| Total for systematic reviews (n, %): | 3 (75) | 4 (100) | 3 (75) |  |  |  |
|  |  |  |  |  |  |  |
| Total for all articles (primary articles, interventions and systematic reviews):** | 33  (31.7) | 82 (78.8) | 56 (53.8) | 81* (81) | 28* (28) |  |

*Note. **Total for all articles for semi-formal and formal sources of support is for primary articles and interventions only as this information was not extracted for systematic reviews.

** Totals and also percentages shown. For access, attitudes and experiences percentages are of the 104 total articles, but for formal and semi-formal sources of support out of the 100 primary articles and interventions. It does not take into account those that were unclear in showing access, attitudes or experiences, or formal or semi-formal sources of support.
