# Supplementary material for: Athletes’ access to, attitudes towards and experiences of help-seeking for mental health: a scoping review
Source: BMJ Open. 2025 Aug 7;15(8):e097492. doi: 10.1136/bmjopen-2024-097492 (PMC12336477; doi:10.1136/bmjopen-2024-097492)
Supplement: Supplementary file 4 [file bmjopen-2024-097492.reviewer_comments.pdf]

PEER REVIEW HISTORY

BMJ Open publishes all reviews undertaken for accepted manuscripts. Reviewers are asked to complete a checklist review form and are provided with free text boxes to elaborate on their assessment. These free text comments are reproduced below.

ARTICLE DETAILS

Title (Provisional)

Athletes' access to, attitudes towards, and experiences of help-seeking for mental health: A scoping review

Authors

Brown, Kirsty R; Quinton, Mary L; Tidmarsh, Grace; Cumming, Jennifer

---

VERSION 1 - REVIEW

**Reviewer**	**1**
**Name**	**Ojio, Yasutaka**
**Affiliation**	**National Center of Neurology and Psychiatry National Institute of Mental Health**
**Date**	**19-Mar-2025**
**COI**	**None**

To Authors:
This study presents a comprehensive and well-executed scoping review on athletes' help-seeking behaviors for mental health support. By employing Rickwood et al.'s help-seeking framework, the study provides a structured categorization of access, attitudes, and experiences, which helps to identify research gaps and inform future studies. The review follows a rigorous methodology, adhering to PRISMA-ScR guidelines, utilizing Covidence for data extraction, and applying Arksey & O'Malley's framework, enhancing its reproducibility and methodological transparency. However, while the study is methodologically sound and theoretically grounded, several areas require further refinement. These include clarifying methodological decisions, addressing theoretical gaps, expanding international comparisons, and better defining key concepts. The following points highlight areas for potential improvement.

Comment 1: The study excludes research that only reports help-seeking percentages, but this decision lacks a clear rationale. While it is understandable that the focus is on more qualitative insights and theoretical contributions, removing such studies may limit epidemiological understanding. Many large-scale surveys provide critical prevalence data on help-seeking behavior, and their exclusion might inadvertently skew the review's conclusions. It would be helpful to explicitly justify this exclusion and discuss its impact on findings.

Comment 2: The study notes that 47.1% of quantitative and mixed-methods studies did not use validated help-seeking measures, yet it does not fully explore how this affects the consistency of findings. Given the diversity of measurement tools used across studies, how were differences in definitions and assessment methods accounted for in the analysis? Additionally, some widely used measures, such as the Attitudes Toward Seeking Professional Psychological Help Scale , focus only on formal help-seeking, potentially underestimating the role of semi-formal or informal support networks. A brief discussion on the limitations

of existing measurement tools and their impact on the comparability of included studies would strengthen the paper.

Comment 3: The study finds that 50% of included studies originate from the US, highlighting a significant geographical bias. While the authors acknowledge the need for more research in low- and middle-income countries, the discussion on how help-seeking behaviors are shaped by different cultural and healthcare contexts remains limited. Given that mental health stigma, access to services, and healthcare policies vary significantly across regions, it would be beneficial to contextualize findings within broader global frameworks. For instance, how do national mental health policies, insurance systems, or societal perceptions of psychological support impact athletes' help-seeking behaviors in different settings?

Comment 4: The study effectively applies Rickwood et al.'s help-seeking framework, which is a major strength. However, additional theoretical perspectives could enhance the depth of the analysis.
The Theory of Planned Behavior (Ajzen, 1991) is briefly mentioned, but its application to help-seeking behaviors could be explored further. Specifically, the role of perceived behavioral control, subjective norms, and intentions in predicting help-seeking could provide valuable insights. Similarly, Self-Determination Theory (Deci & Ryan, 2000) could help explain why athletes are more or less likely to seek help based on the level of autonomy-supportive vs. controlling environments in their teams and organizations.

Comment 5: The study treats attitudes toward help-seeking as a broad, undifferentiated construct, but prior research suggests that self-stigma, public stigma, anticipated negative consequences, and personal willingness are distinct components influencing help-seeking. A more nuanced approach to categorizing attitudes—for example, separating cognitive (beliefs about help-seeking), affective (emotional responses), and behavioral (intentions to seek help) components—would enhance conceptual clarity and improve alignment with existing psychological models.

Comment 6: The study rightly identifies the lack of research from low- and middle-income countries, but Asian research is particularly underrepresented despite the growing interest in athlete mental health in the region. Japan, for example, has made progress in understanding how masculine norms shape help-seeking behavior among male athletes, particularly in rugby and other high-contact sports. Studies on mental health literacy, alternative approaches to mental conditioning, and culturally adapted interventions provide valuable insights that could complement the review's findings. I encourage the authors to incorporate relevant studies from Japan and other Asian countries to strengthen the international perspective of the review. This would help move beyond Western-centric frameworks and offer a more holistic understanding of global help-seeking behaviors.

Comment 7: The review finds that only 2% of included studies examine help-seeking from semi-formal sources, such as coaches, team leaders, or academic advisors. This is a significant gap, as emerging evidence suggests that athletes often seek support from familiar figures before engaging with formal mental health services. Studies on peer-led mental health support, mentorship programs, and coach training in psychological first aid could provide useful directions for future research. Strengthening the discussion on how semi-formal support networks can be leveraged to improve help-seeking would be a valuable addition.

Comment 8: The study highlights that many interventions focus on attitudes rather than access, but practical recommendations for improving access remain limited. It would be useful to outline concrete strategies for improving help-seeking pathways, such as: Embedding mental health resources within athletic organizations;
Providing mental health first aid training for coaches and team leaders; Leveraging digital tools (e.g., mental health apps, online consultations) to improve accessibility. A brief discussion on how findings could inform policy and practice would significantly enhance the review’s applied relevance.

**Reviewer**	**2**
**Name**	**Montero, Ashley**

**Affiliation**	**Flinders University**
**Date**	**04-Apr-2025**
**COI**	**None**

Thank you for providing me an opportunity to review this manuscript on help seeking behaviours in athletes. The authors have addressed the majority of comments highlighted from previous revision to a satisfactory standard; here, I have included my additional feedback and comments to further improve the quality of this manuscript.

Comments

P2 L27: Sentence should finish with "of studies".

P3 L47-48: Incomplete sentence.

P6: The authors have not included a seminal systematic review by Cosh et al. (2014) - https://doi.org/10.1016/j.psychsport.2023.102586 . This was one of the 4 SLR's from the literature review, and the only one not to be discussed. This is an important review in the field and given the rigour of the methodology and protocol, it is confusing that this was not included.

Table 4: The headings were hard to navigate. 'Future research direction recommended' and 'evidence from this study' should be able 'overview of included studies'

Table 4: Spelling error "This research needs must specify…"

Table 4: the last two recommendations for overview of included studies should be in separated rows.

Discussion: The discussion feels a little underdone regarding the connection between literature and summative findings from the scoping review.

Limitations and future research directions: The authors acknowledge that they only reviewed papers that were published in English, but emphasise the disparity of research in the USA and 'westernised nations'. While there is certainly a disparity here, this may be partially owing to the selection of articles reviewed. Similarly, greater reference to the importance of research in culturally diverse populations who do not fit the WEIRD (Western, Educated, Industrialized, Rich and Democratic) criteria should be made.

VERSION 1 - AUTHOR RESPONSE

Reviewer: 1

Comment:	*Response:*
This study presents a comprehensive and well-executed scoping review on athletes' help-seeking behaviors for mental health support.	Thank you for your positive comments about our scoping review. We would like to thank you for your detailed comments and suggested

By employing Rickwood et al.'s help-seeking framework, the study provides a structured categorization of access, attitudes, and experiences, which helps to identify research gaps and inform future studies. The review follows a rigorous methodology, adhering to PRISMA-ScR guidelines, utilizing Covidence for data extraction, and applying Arksey & O'Malley's framework, enhancing its reproducibility and methodological transparency. However, while the study is methodologically sound and theoretically grounded, several areas require further refinement. These include clarifying methodological decisions, addressing theoretical gaps, expanding international comparisons, and better defining key concepts. The following points highlight areas for potential improvement.	refinements as they were really helpful in improving the review further.
Comment 1:<br><br>The study excludes research that only reports help-seeking percentages, but this decision lacks a clear rationale. While it is understandable that the focus is on more qualitative insights and theoretical contributions, removing such studies may limit epidemiological understanding. Many large-scale surveys provide critical prevalence data on help-seeking behavior, and their exclusion might inadvertently skew the review's conclusions. It would be helpful to explicitly justify this exclusion and discuss its impact on findings.	*Response 1:*<br><br>Thank you for your comment on the exclusion of papers looking at help-seeking percentages.<br><br>We feel that the second sentence of the introduction may have been misleading and therefore we have replaced it with: "What is less clear is the current state of the literature that encapsulates the perspective of athletes on different stages of the mental health help-seeking process." (p.4, line 76).<br><br>We provided a rationale in supplementary file 1: *"Papers that just included data on the percentage of athletes that sought help were excluded. For example, a paper saying that "20% of athletes sought help for their mental health" does not tell us anything about access, attitudes or experiences in relation to mental health help-seeking"* which we have now moved into the main manuscript (p.7, line 222) as we agree that this explanation is important to include.<br><br>We would like to point out that this specific exclusion criteria did not apply to many studies, and when it did, these studies tended to have small samples. Epidemiological research in athlete mental health is very limited, and so these help-seeking percentages in smaller scale studies will not give us the population level

	insights that epidemiological research aims to achieve.<br><br>Regardless of this issue, we recognize a need for systematic review on help-seeking percentages. But this is beyond the scope of ours and warrants its own review to adequately assess the percentages as well as quality of these studies. We have added this point as a further research direction (p.18).
Comment 2a:<br><br>The study notes that 47.1% of quantitative and mixed-methods studies did not use validated help-seeking measures, yet it does not fully explore how this affects the consistency of findings. Given the diversity of measurement tools used across studies, how were differences in definitions and assessment methods accounted for in the analysis?	*Response 2a:*<br><br>Thank you for your comment, and we agree that the impact of validated measures on the findings would benefit the analysis.<br><br>We have added an additional column in appendix 6 to capture validated measures, now highlighting which studies used validated versus non-validated measures.<br><br>We do not feel we can accurately analyse the impact of validated measures on the consistency of findings as this is outside of the scope of the review. However, validated measures tend to be more be more explicit in what they measured, and therefore we decided that this pattern analysis would allow us to reflect on this issue more accurately within the manuscript. For example, as you note, papers that used the validated measure of Attitudes Toward Seeking Professional Psychological Help Scale focuses only on formal help-seeking.<br><br>We have also carried out a pattern analysis for validated vs non-validated measures for the mixed methods and quantitative studies, by formal and semi-formal, and access, attitudes, and experiences. This is shown in tables 5 and 6 which we calculated in appendix 6 and also discussed within the manuscript (p.13, line 410).<br><br>We agree that the analysis of the impact of validated measures on the consistency of findings in the athlete help-seeking literature would be of benefit and therefore added a point within the further research directions recommending the need for a systematic review on help-seeking measures used within

	the athlete mental health help-seeking literature (p.17).
Comment 2b:<br><br>Additionally, some widely used measures, such as the Attitudes Toward Seeking Professional Psychological Help Scale, focus only on formal help-seeking, potentially underestimating the role of semi-formal or informal support networks. A brief discussion on the limitations of existing measurement tools and their impact on the comparability of included studies would strengthen the paper.	*Response 2b:*<br><br>Thank you for raising this point. Following on from the pattern analysis, we have added a discussion point highlighting that validated measures focus on attitudes and formal sources of support (p.16, line 477).<br><br>We have also added a future research direction stating the need for validated measures on help-seeking from semi-formal sources of support, that encapsulate views on access and experiences (p.19-20).
Comment 3:<br><br>The study finds that 50% of included studies originate from the US, highlighting a significant geographical bias. While the authors acknowledge the need for more research in low- and middle-income countries, the discussion on how help-seeking behaviors are shaped by different cultural and healthcare contexts remains limited. Given that mental health stigma, access to services, and healthcare policies vary significantly across regions, it would be beneficial to contextualize findings within broader global frameworks. For instance, how do national mental health policies, insurance systems, or societal perceptions of psychological support impact athletes' help-seeking behaviors in different settings?	*Response 3:*<br><br>Thank you for raising this point. We have added a sentence and relevant references to identify the various factors across countries that may impact athlete help-seeking behaviours (p.16, line 450).<br>We have also added to the directions for future research (p.18).
Comment 4:<br><br>The study effectively applies Rickwood et al.'s help-seeking framework, which is a major strength. However, additional theoretical perspectives could enhance the depth of the analysis.<br>The Theory of Planned Behavior (Ajzen, 1991) is briefly mentioned, but its application to help-seeking behaviors could be explored further. Specifically, the role of perceived behavioral control, subjective norms, and intentions in predicting help-seeking could provide valuable	Response 4:<br><br>Thank you for recognizing the strength of utilizing Rickwood et al.'s help-seeking frameworks. The use of additional perspectives is a valid point, however since such little theory has been used in the primary research we extracted, we decided to focus on Rickwood et al.'s frameworks with depth throughout the review and its analysis.<br><br>Although it was beyond the scope of the review to analyze the use of help-seeking frameworks

insights. Similarly, Self-Determination Theory (Deci & Ryan, 2000) could help explain why athletes are more or less likely to seek help based on the level of autonomy-supportive vs. controlling environments in their teams and organizations.	in detail, we have done additional analysis of papers in terms of theory used, and how it has been used in the included studies, where appropriate (can be seen in appendix 6). We have added a paragraph to the manuscript to summarise these findings (p.15, line 429).<br><br>We agree that the use of additional frameworks beyond Rickwood et al.'s should be used within the help-seeking literature going forwards, particularly in interventions. For example, the theory of planned behaviour and self-determination theory, as well as Rickwood and colleagues should be better utilised in the athlete mental health literature. We have added a point under future research directions to highlight the need for the use of frameworks in the literature going forwards (p.20).
Comment 5:<br><br>The study treats attitudes toward help-seeking as a broad, undifferentiated construct, but prior research suggests that self-stigma, public stigma, anticipated negative consequences, and personal willingness are distinct components influencing help-seeking. A more nuanced approach to categorizing attitudes—for example, separating cognitive (beliefs about help-seeking), affective (emotional responses), and behavioral (intentions to seek help) components—would enhance conceptual clarity and improve alignment with existing psychological models.	*Response 5:*<br><br>Thank you for raising this point on the definition of attitudes. We do recognize that attitudes encompasses many different aspects, which we hope to have shown in the definition that we have used. Owing to the nature of the scoping review, and the lack of consistent definitions used across papers, we formed these definitions as we carried out the review, in order to make the analysis of the papers more accurate. However, we hope this is a starting point in the literature to address these definitional issues.<br><br>With respect to taking a more nuanced approach to attitudes, we have re-analysed the quantitative and mixed methods papers that use validated measures to provide a more detailed analysis of attitudes, using the help-seeking validated measures that they have used.<br>We have selected based on general attitudes, stigma, preferences/expecations for support, and behavioural intentions to seek help. The results are available in table 3 (p.13).
Comment 6:<br><br>The study rightly identifies the lack of research from low- and middle-income countries, but Asian research is particularly underrepresented despite the growing interest in athlete mental	*Response 6:*<br><br>Thank you for identifying this important point. We have added to the discussion to highlight the lack of studies from Asian countries (p.16,

health in the region. Japan, for example, has made progress in understanding how masculine norms shape help-seeking behavior among male athletes, particularly in rugby and other high-contact sports. Studies on mental health literacy, alternative approaches to mental conditioning, and culturally adapted interventions provide valuable insights that could complement the review's findings. I encourage the authors to incorporate relevant studies from Japan and other Asian countries to strengthen the international perspective of the review. This would help move beyond Western-centric frameworks and offer a more holistic understanding of global help-seeking behaviors.	line 451), referencing 2 that were included in the review.<br>We have also added a future research direction on the need for further studies on Asian countries (p.18).
Comment 7:<br><br>The review finds that only 2% of included studies examine help-seeking from semi-formal sources, such as coaches, team leaders, or academic advisors. This is a significant gap, as emerging evidence suggests that athletes often seek support from familiar figures before engaging with formal mental health services. Studies on peer-led mental health support, mentorship programs, and coach training in psychological first aid could provide useful directions for future research. Strengthening the discussion on how semi-formal support networks can be leveraged to improve help-seeking would be a valuable addition.	*Response 7:*<br><br>Thank you for this suggestion. We have added to the discussion to highlight the need for leveraging semi-formal sources of support for athletes (p.17, line 510).
Comment 8:<br><br>The study highlights that many interventions focus on attitudes rather than access, but practical recommendations for improving access remain limited. It would be useful to outline concrete strategies for improving help-seeking pathways, such as: Embedding mental health resources within athletic organizations; Providing mental health first aid training for coaches and team leaders; Leveraging digital tools (e.g., mental health apps, online consultations) to improve accessibility. A brief discussion on how findings could inform policy and practice would significantly enhance the review's applied relevance.	*Response 8:*<br><br>Thank you, we have added to the conclusion to outline policy and practice implications of this review, and provided a few concrete strategies (p.21, line 550).

Reviewer: 2

Comment:	Response:
Thank you for providing me an opportunity to review this manuscript on help seeking behaviours in athletes. The authors have addressed the majority of comments highlighted from previous revision to a satisfactory standard; here, I have included my additional feedback and comments to further improve the quality of this manuscript.	Thank you for reviewing our manuscript and for the additional feedback which we believe has served to improve the manuscript further.
Comment 1:<br><br>*P2 L27: Sentence should finish with "of studies".*<br>*P3 L47-48: Incomplete sentence.*	*Response 1:*<br><br>Thank you for bringing these to our attention. We have now addressed these in the manuscript as follows:<br><br>access to sources of support in 32.2% of studies. (p.2, line 48)<br><br>For example, athletes may fear that disclosure and help-seeking may have a negative impact upon their selection to a team/squad (p.4, line 91).
Comment 2:<br><br>P6: The authors have not included a seminal systematic review by Cosh et al. (2014) - https://doi.org/10.1016/j.psychsport.2023.102586 . This was one of the 4 SLR's from the literature review, and the only one not to be discussed. This is an important review in the field and given the rigour of the methodology and protocol, it is confusing that this was not included.	*Response 2:*<br><br>Thank you for this comment. The systematic review by Cosh et al. (2024) is one of the four reviews that was located and included during the scoping review searches.<br><br>In the first paragraph of the rationale in the introduction, we only discuss two of the included systematic reviews (Moreland, Cox and Yan, 2018; Castaldelli-Maia et al., 2019) due to word limits. However, we did mistakenly miss out the reference for Cosh et al., (2024), and this has now been updated to include all 4 systematic reviews.<br><br>Within the synthesis of the results section, we state that "and the 4 systematic reviews were published in 2018, 2019, 2020 and 2024" although the reference for the Cosh et al., (2024) paper was missing so this has now been updated.

	The details of extractions are available in appendix 6, and within supplementary file 10 under the subheading of the table "systematic reviews".
Comment 3:<br><br>Table 4: The headings were hard to navigate. 'Future research direction recommended' and 'evidence from this study' should be able 'overview of included studies'<br>Table 4: Spelling error "This research needs must specify…"<br>Table 4: the last two recommendations for overview of included studies should be in separated rows.	*Response 3:*<br><br>Thank you, we have removed the 'Future research direction recommended' and 'evidence from this study' subheadings and just left it as overview of included studies. I have added subheadings within the table to clearly outline the future research directions. I have added "and evidence from this review" in the title of the table. (p.18)<br><br>We have corrected the spelling error: The research needs to…. specify race or ethnicity… (p.19)<br><br>I have now put the last two recommendations for overview of included studies in separate rows (p.19).
Comment 4:<br><br>Discussion: The discussion feels a little underdone regarding the connection between literature and summative findings from the scoping review.	*Response 4:*<br><br>Thank you for your comment. Given the feedback from both reviewers' on the discussion, we believe the additions have now strengthened the connection between the literature and summative findings from the review (p.16-18).
Comment 5:<br><br>Limitations and future research directions: The authors acknowledge that they only reviewed papers that were published in English, but emphasise the disparity of research in the USA and 'westernised nations'. While there is certainly a disparity here, this may be partially owing to the selection of articles reviewed. Similarly, greater reference to the importance of research in culturally diverse populations who do not fit the WEIRD (Western, Educated, Industrialized, Rich and Democratic) criteria should be made.	*Response 5:*<br><br>Thank you for highlighting this point.<br>Thank you for identifying this important point alongside reviewer 1.<br>We have added to the discussion to highlight the lack of studies from Asian, African and Latin American countries (p.16, line 447).<br>We have also added a future research direction on the need for further studies from these countries (p.18).
